# Phased-array sources based on nonlinear metamaterial nanocavities

**DOI:** 10.1038/ncomms8667

**Published:** 2015-07-01

**Authors:** Omri Wolf, Salvatore Campione, Alexander Benz, Arvind P. Ravikumar, Sheng Liu, Ting S. Luk, Emil A. Kadlec, Eric A. Shaner, John F. Klem, Michael B. Sinclair, Igal Brener

**Affiliations:** 1 Center for Integrated Nanotechnologies, Sandia National Laboratories, PO Box 5800, Albuquerque, New Mexico 87185, USA; 2 Science & Technology Division, Sandia National Laboratories, PO Box 5800, Albuquerque, New Mexico 87185, USA; 3 Department of Electrical Engineering, Princeton University, Princeton, New Jersey 08544, USA

## Abstract

Coherent superposition of light from subwavelength sources is an attractive prospect for the manipulation of the direction, shape and polarization of optical beams. This phenomenon constitutes the basis of phased arrays, commonly used at microwave and radio frequencies. Here we propose a new concept for phased-array sources at infrared frequencies based on metamaterial nanocavities coupled to a highly nonlinear semiconductor heterostructure. Optical pumping of the nanocavity induces a localized, phase-locked, nonlinear resonant polarization that acts as a source feed for a higher-order resonance of the nanocavity. Varying the nanocavity design enables the production of beams with arbitrary shape and polarization. As an example, we demonstrate two second harmonic phased-array sources that perform two optical functions at the second harmonic wavelength (∼5 μm): a beam splitter and a polarizing beam splitter. Proper design of the nanocavity and nonlinear heterostructure will enable such phased arrays to span most of the infrared spectrum.

## Abstract (web summary)

Controlling light at scales smaller than its wavelength is attractive to manipulate light using small device footprints. Here, the authors propose a scheme to modify light on such small scales using a combination of metamaterial nanocavities coupled to nonlinear semiconductor heterostructures.

## Graphical abstract

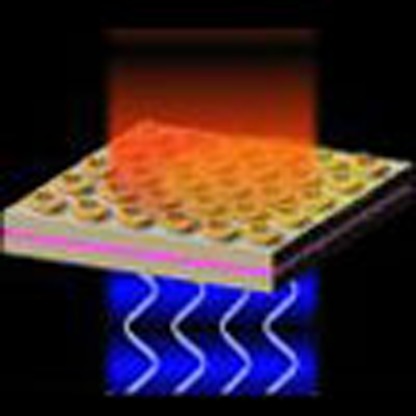

Phased antenna arrays comprise ensembles of subwavelength sources, each radiating with a definite phase relationship relative to the other elements of the array^1^,^2^. They are widely used in the microwave region of the spectrum^3^,^4^,^5^,^6^,^7^, where applications such as beam scanning can be achieved by appropriately choosing the phase of the excitation fed to each of the elements in the array. Such phased-array sources are also appealing for use at infrared wavelengths^8^ for applications such as beam shaping and steering, and polarization conversion. However, translating these radio-frequency concepts to higher frequencies becomes problematic since isolated feed lines do not exist for each of the array elements, which prohibits arbitrary excitation of the array. To overcome this limitation, researchers have employed specially tailored planar metamaterial or nanoantenna arrays which, on uniform excitation using plane waves, re-radiate energy with desired spatial and polarization characteristics^9^,^10^,^11^,^12^. In this approach, the design of the individual resonator is varied across the array to achieve the desired phase and polarization response—in essence, the elements of the array also acquire the role of the phase shifters and polarization converters. These types of plasmonic-nanoantenna arrays have been successfully used to control the phase front^9^,^10^ and spatial polarization^11^,^12^ of an incident beam, while having a very small (subwavelength) footprint and being able to operate over wide frequency ranges. In practice, however, such an approach cannot achieve 100% conversion efficiency and one is forced to contend with unconverted remnants of the excitation beam in the output. More recently, this concept has been extended to include subwavelength ‘Huygens sources'^13^,^14^,^15^ as the radiating elements within the array. Huygens' sources combine electric dipole and magnetic dipole behaviour to improve the input/output conversion efficiency of the array.

We propose a new concept for phased-array sources at infrared wavelengths that makes use of localized nonlinear polarization to create a phase-locked ‘feed' to subwavelength metamaterial nanocavities which re-radiate a beam with desired spectral, spatial and polarization properties. Our approach utilizes arrays of doubly resonant, metallic, metamaterial nanocavities that are near-field coupled to highly nonlinear intersubband transitions (ISTs) in semiconductor quantum wells. Strong coupling of metamaterial resonators to ISTs has recently been demonstrated^16^,^17^, which has enabled efficient access to the giant nonlinearities offered by ISTs^18^,^19^. Another phased approach using the intrinsic nonlinearities of metamaterial photonic crystals was recently investigated^20^. In this work, we show that a device (graphically depicted in Fig. 1) based on split ring resonator (SRR) nanocavities and having quantum wells as the nonlinear medium allows for complete control over the polarization of an emitted second harmonic (SH) signal. We then demonstrate that the resonators emit spatially phase-coherent SH radiation, and finish by combining these two results to demonstrate a phased-array design that controls the far-field angular distribution of the SH. Near-term applications based on static versions of the proposed device are shown in the insets of Fig. 1. In the longer term, we envision that electrically tuning the quantum well levels or nonlinearity will allow real-time control over the output beam characteristics.

## Results

### Operating principle and resonator design

**Figure 2:**
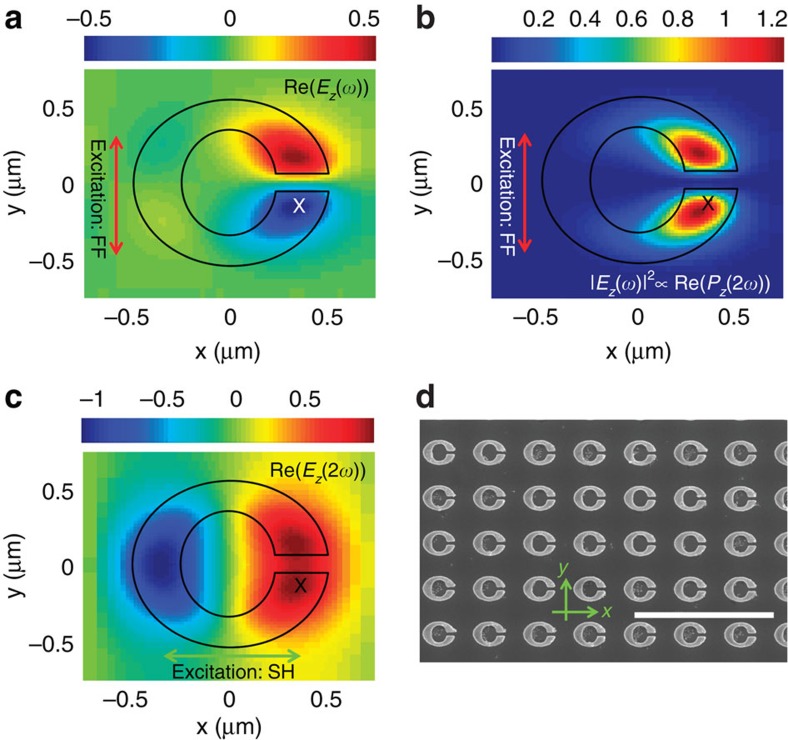
Resonator design. (**a**) Real part of the *z*-component of the electric field, *E*_*z*_, at the FF. (**b**) Squared magnitude of *E*_*z*_ at the FF, which is proportional to the nonlinear polarization. (**c**) Real part of *E*_*z*_ at the SH frequency. The maps in (**a**–**c**) are computed within the quantum well, at a depth of 150 nm below the resonator in a unit cell simulated with FDTD full-wave simulations. The black/white X marks the position of the plot in Fig. 3b. (**d**) SEM image of the fabricated nanocavities on the sample surface. Scale bar, 4 μm.

Figure 2 presents the basic operating principle of our device. The SRRs are optimized to support two, cross-polarized resonances that match the desired fundamental frequency (FF) and SH frequency. The fundamental resonance is *y*-polarized (vertical in Fig. 2) and occurs at ∼113 meV, while the SH resonance is *x*-polarized (horizontal) at ∼226 meV (resonators' dimensions and metamaterial periods are given in the Methods section). For the moment, we ignore the details of the nonlinear quantum well medium, except to stress that accessing the nonlinearities of the ISTs requires a *z*-directed electric field within the quantum well multilayer. We perform linear full-wave finite-difference-time domain (FDTD) simulations^21^ to examine the electric field distributions under plane wave excitation at the fundamental and SH resonances. The real part of the *z*-directed electric field in a plane located 150 nm below the SRR resulting from excitation at the fundamental resonance is shown in Fig. 2a. Note that the field is highly localized in the vicinity of the SRR gap and is anti-symmetric with respect to the middle of the gap. Owing to the large IST nonlinearity, this field induces a localized, phase-locked polarization at the SH frequency, 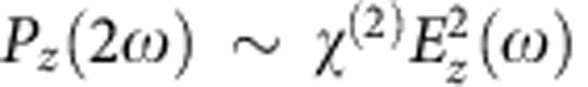. Figure 2b shows the profile of the square of the fundamental electric field below the SRR, which is proportional to the nonlinear polarization. The nonlinear polarization is localized in the vicinity of the gap and is symmetric with respect to the gap center. The real part of the *z*-directed electric field at the SH resonance of the SRR and located in a plane 150 nm below the SRR is shown in Fig. 2c. Like the nonlinear polarization, the SH field is symmetric with respect to the gap centre. Comparing Fig. 2b,c reveals that the nonlinear polarization acts as a SH feed source that excites the SH mode of the SRR. The excited SH mode is then capable of re-radiating at the SH frequency in a manner that is phase coherent with the other resonators in the array. Since the SH emission is largely dipolar in character, the array will generally emit in both the forward and backward directions. Figure 2d shows a scanning electron microscopy (SEM) image of part of the 1-mm^2^ array of the fabricated SRRs, which were patterned on the semiconductor heterostructure using electron beam lithography. Linear transmission simulations and measurements of the fabricated sample are discussed in Supplementary Note 1 and depicted in Supplementary Fig. 1.

### Nonlinear medium design

**Figure 3:**
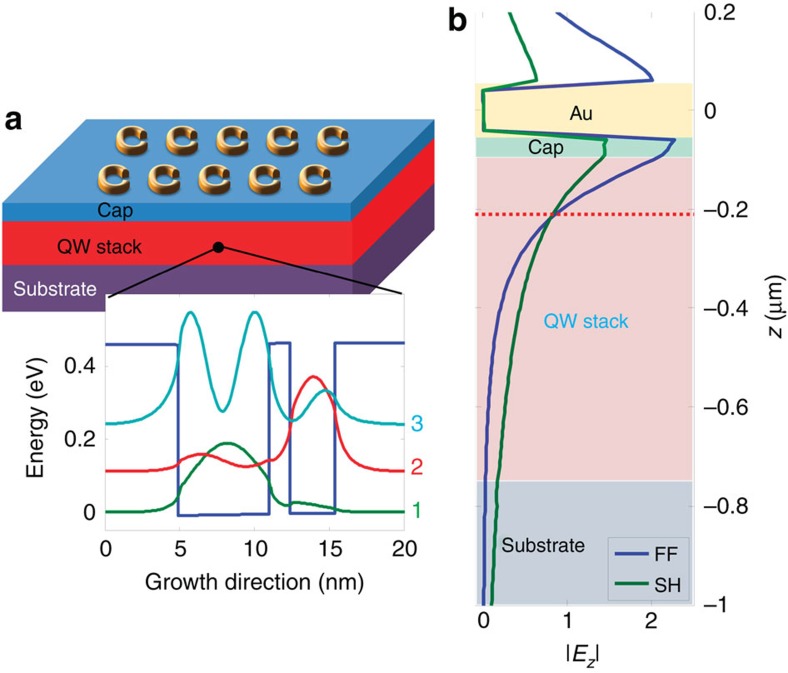
Quantum well design. (**a**) Schematic representation of the device, gold nanoresonators are patterned on top of the III–V multi-quantum well structure. (inset) Calculated band structure for one period of the quantum well heterostructure, the blue line depicts the conduction band, the green, red and light blue curves represent the probability density distributions for the first, second and third intersubbands, respectively, shifted according to their energies. (**b**) |*E*_*z*_| as a function of depth into the quantum well stack at the position marked with an X symbol in Fig. 2, for FF and SH frequencies. We note a good field overlap that enables efficient SH generation. The red dotted line represents the position of the field plots from Fig. 2a–c.

To optimize the nonlinear response of the quantum wells, we use a design that is resonant at both the fundamental and SH frequencies. Figure 3a shows the band structure and resulting wavefunctions/eigenenergies for one period of the quantum well^22^. Specifically, the design features three subband energy levels that are equally separated by ∼125 meV. Thus, the transitions between these levels occur with energies of ∼125 meV (∼10 μm) for the 1→2 and 2→3 transitions (1 being the ground state shown with a green line in Fig. 3a; 2 the first excited state corresponding to the red line; and 3 the second excited state represented by a light blue line). Such an arrangement results in a 3→1 transition energy of ∼250 meV (∼5 μm). The giant second-order nonlinear resonant susceptibility *χ*^(2)^ for this process is given by^23^: 
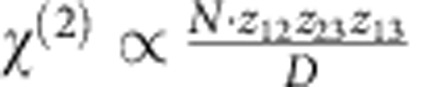
 where *N* is the carrier density, *z*_*ij*_ is the transition dipole moment between levels *i* and *j*; *D* denotes an expression that is minimized and becomes real when the three energy levels are equally spaced^23^ and the level separations are equal to the incident photon energy. Note that the measured transition energies of the fabricated quantum wells occurred at 113 and 226 meV, and the SRRs were subsequently designed to match these energies. More details on the heterostructure and experimental observation of the ISTs are given in the Methods section and Supplementary Note 2 and Fig. 2. Figure 3b shows the penetration of the *z*-directed fundamental electric field into the multi-quantum well layer.

### SHG characterization

**Figure 4:**
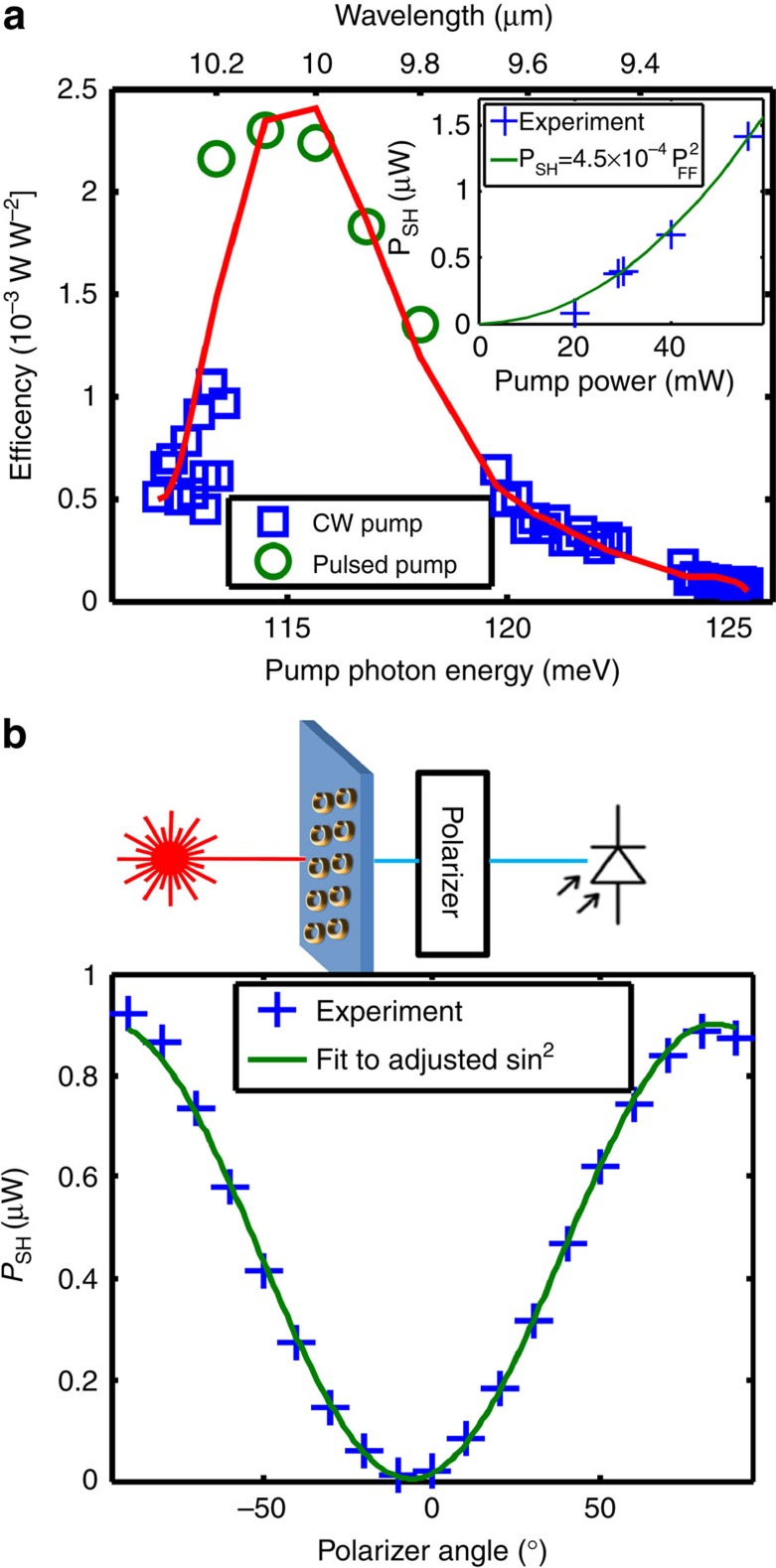
SH generation characterization. (**a**) SH conversion efficiency as a function of pump wavelength. The inset shows the SH power dependence on pump power for a pump wavelength of 10.22 μm and fit to a quadratic function with *η*=0.45 mW W^−2^. The red line is a guide to the eye. (**b**) SH signal as a function of polarizer angle. Polarizer angle of 0° corresponds to the pump polarization and the fitting function is adjusted for unintentional sample tilt. The inset shows a schematic of the experimental setup for the polarization measurement.

The inset of Fig. 4a shows the dependence of the measured SH power on the fundamental power for wavelength of 10.22 μm (blue crosses) and a quadratic fit 
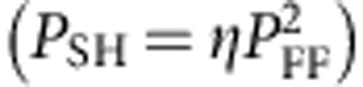
 to the data (solid green). From this fit we extract an experimental SH conversion efficiency of 0.45 mW W^−2^. FDTD simulations for these experimental conditions predict a larger efficiency of 1.9 mW W^−2^. This discrepancy is likely due to imperfections in fabrication, an imprecise estimate for the value of *χ*^(2)^, and pump beam profile imperfections. To highlight the spectral response of our system, which depends resonantly on both the SRRs and quantum wells, we performed power-dependence measurements with varying pump wavelengths. We plot the conversion efficiency, *η*, as a function of pump frequency in Fig. 4a. The efficiency has a strong dependence on wavelength and changes by more than an order of magnitude (from ∼0.08 to ∼2.3 mW W^−2^) when the photon energy is varied by 10 meV (∼800 nm at these wavelengths). Interestingly, we obtain conversion efficiencies as high as ∼2.3 mW W^−2^—the highest value reported so far in metamaterial-IST coupled structures. Taking into account Fresnel reflections of the SH at the exit substrate–air interface and the prediction in ref. 18 for equal SH power generated in reflection, the overall conversion efficiency is ∼5.3 mW W^−2^. This efficiency value is quite remarkable considering the length of our active medium is less than a fifth of the SH wavelength. Theoretical predictions claim that a conversion factor of 50% is possible for a second-order resonant process^24^, so in principle our design could be further optimized to approach such predictions.

The data of Fig. 4a were obtained using both pulsed and a continuous wave sources indicating that our device works with both configurations. However, for high pump intensities (>6 kW cm^−2^) we observe a cross-over to linear dependence with a constant conversion factor of ∼0.14% (more details in Supplementary Note 3 and Fig. 3) or, when taking into account the corrections mentioned above, ∼0.36%.

Using a polarizer located after the sample, as shown at the top of Fig. 4b, we measured the SH signal as a function of the polarizer angle with respect to the pump polarization. The measured data are plotted as blue crosses in Fig. 4b, and are well fitted by a squared sine function (green line). Since the signal is maximized near *θ*=±90°, we conclude that the SH signal is cross-polarized to the pump, as expected. More details about the polarization experiments are reported in Supplementary Note 4 and Fig. 4. The polarization experiments reveal that we can describe the nonlinear response of our device with an effective susceptibility containing only one component 
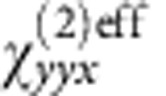
, where *y* represents the excitation polarizations and *x* the output polarization. We note that this behaviour arises due to the resonator design and, thus, arbitrary spatial polarization profiles of the SH beam could be achieved by selectively rotating some of the resonators and illuminating with circular or 45° polarization as demonstrated below.

### Coherence demonstration

**Figure 5:**
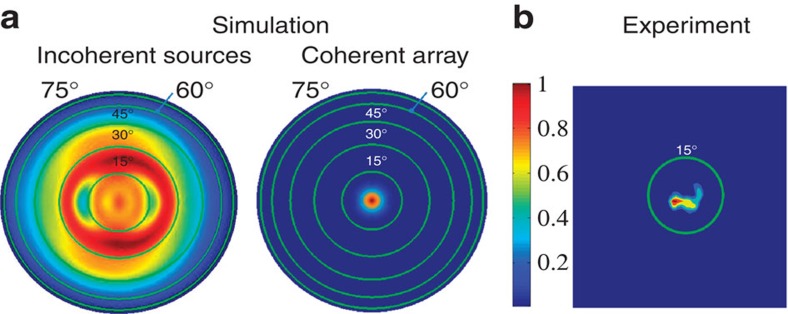
Coherence. (**a**) Simulated, normalized far-field hemispherical intensity plot for incoherent (left) and phase-coherent (right) emission from the array. The centre corresponds to radiation in the zenith direction while the green circles mark the polar angles of 15° (inner circle) to 75° (outer circle) in 15° increments from the zenith. (**b**) Experimental far-field pattern, where the green circle represents the max polar angle measured (∼15°).

To assess the spatial coherence of the resonators, we simulated the expected radiation pattern of the array for two distinct scenarios: in the first case the nanocavities emit incoherently; and in the second case the nanocavities are coherent and the relative phase of the emitted radiation is determined by the FF pump. In the left half of Fig. 5a we plot the (normalized) simulated hemispherical intensity pattern for the first case. This was obtained by first computing the far-field pattern of a single nanocavity and then summing intensities from all contributing radiators. As seen in the figure, the intensity for this case is fairly uniform over the angles accessible in our measurement setup (∼15°, corresponding to the innermost circle in Fig. 5a). The simulated far-field intensity pattern for the second case is plotted in the right half of Fig. 5a. In this case we use the ‘array factor' from antenna theory^1^ by summing the individual contributions of elements in a finite array, where each element is weighted according to their relative illumination intensity from the pump beam. The resulting radiation pattern consists of a strong lobe at the zenith of the hemisphere. The experimentally measured far-field pattern, presented in Fig. 5b, clearly shows a narrow lobe reminiscent of the second simulated case. This measurement was performed using a spot size of ∼100 μm and the lasing line (10.247 μm) was selected as to have a nominal spatial mode of TEM_00_. Under these conditions, the pump power at the sample plane was ∼56 mW. The measured radiation exhibits an angular divergence of about 3° (in the narrow dimension), which is very similar to the calculated divergence of 4°. The small deviations of the lobe angle and width from numerical predictions can be explained by imperfections of the pump intensity profile, as well as fabrication imperfections that might result in a variation of resonator shape across the beam. Nevertheless, the results in Fig. 5 suggest that all the resonators are acting as a collection of phase-coherent sources of SH radiation. We have also simulated the second case for incident beam waists smaller than the beam waist used in our experimental setup. This corresponds to an intermediate case where the coherence length is smaller than the experimental beam's spot size (Supplementary Fig. 5). We observe that the lobe width grows quickly as the beam waist decreases, further confirming our observation that the entire radiating array is coherent. More details on the far-field calculations are reported in Supplementary Note 5.

### Beam manipulation

**Figure 6:**
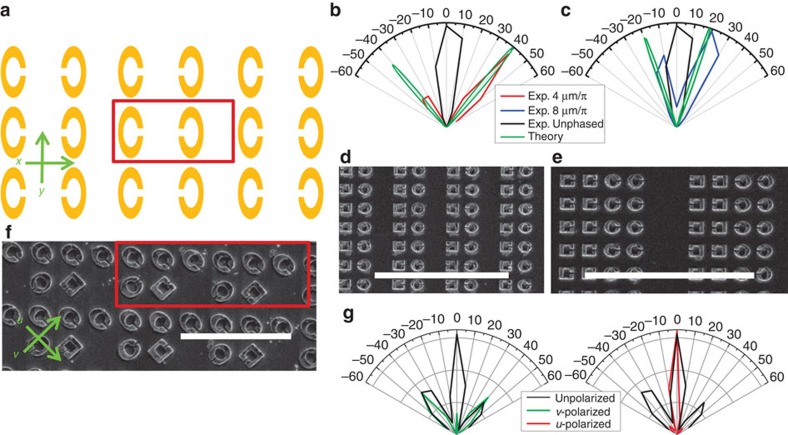
Beam manipulation. (**a**) Schematic of an array designed for beam splitting. The unit cell is marked by the red rectangle. The phase variation is in the *x*-direction. This design has one element per phase step. (**b**,**c**) Normalized simulation and experimental far-field intensity in the *x–z* half plane for the samples shown in the SEM micrographs in (**d**,**e**). Scale bar, 10 μm. (**f**) SEM of a fabricated metasurface designed as a ‘polarizing beam splitter'; a unit cell is designated by the red rectangle. Scale bar, 5 μm. (**g**) Normalized experimental measurement for the sample depicted in (**f**) for two orthogonal polarizations of the SH radiation when the illuminating FF beam is polarized along the *y* axis. The legend denotes the analyser polarization.

The above results imply that by varying the resonator shape across the sample, as was first done in ref. 9 for degenerate wavefront manipulation, one can create a source with an arbitrary wavefront. To demonstrate this and to further emphasize that all the metamaterial resonators radiate in a phase-coherent manner, we present in Fig. 6a a schematic example of an array design that splits the SH radiation into two beams with a predetermined angular separation. This design uses the π phase shift that results from mirroring the resonator with respect to the *y* axis (this is elaborated in Supplementary Note 6). Figure 6b (Fig. 6c) shows simulations and experimental data of the far-field radiation pattern from two designs based on this principle but consisting of two (four) resonators for each orientation for a four (eight)-resonator unit cell, as depicted in the SEM micrograph in Fig. 6d (Fig. 6e). Through full-wave simulation, we have determined that the square resonators shown in the micrographs radiate with the same phase as the elliptical resonators. We observe radiation lobes at ∼±40° (∼±20°) as expected for the 5.124-μm wavelength and 8-μm (16-μm) periodicity of the design. Good agreement between the measurements and simulations is observed. In this periodic design, the angle, *θ*, of the emitted radiation (with respect to the broadside) is determined by the overall periodicity of the array, and the distribution of radiation among the lobes is controlled by the ‘phase slope' 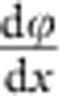 within the unit cell. In the simple implementation shown here we do not break the symmetry between positive and negative ‘phase slope', so similar intensities are observed in both lobes. By adding additional resonators that radiate with different phases, this right–left symmetry can be broken and the magnitude of one of the lobes can be reduced. In general, the phase profile does not need to be periodic, and any arbitrary radiation pattern can be generated if the correct phase profile^25^ is used.

Figure 6f depicts a micrograph of a metasurface designed to combine polarization control together with the beam-splitting capability, the red rectangle marks a unit cell. On illumination with *y*-polarized FF light, the array radiates a single beam in the broadside direction polarized along the *u* axis (45° with respect to the *y* axis) and two beams at ∼±40° polarized along the *v* axis (−45° with respect to the *y* axis). The *u*-polarized broadside beam is generated by the top row of resonators in the unit cell, where the periodicity in *x* was minimized to: (a) increase resonator density thereby increasing the generated SH intensity and (b) decrease the angular width of the radiated lobe. The two *v*-polarized beams originate from the resonators in the bottom row of the unit cell, where the periodicity in *x* was selected to generate beams at ∼±40°. In Fig. 6g, we report the experimental far-field pattern obtained by measuring unpolarized radiation and radiation polarized along the *u-* and *v*-directions. The results agree very well with theoretical predictions and there is very little crosstalk between the two polarizations.

## Discussion

We have demonstrated a new approach to phased-array sources at mid-infrared wavelengths that utilizes nonlinear optical processes to create a phase-locked, localized feed to the resonators of the array. Although the examples presented in this manuscript focused on SH generation, the new concept is general and can be applied to other types of nonlinear frequency generation. For example, large resonant third-order nonlinear susceptibilities have also been demonstrated in quantum wells^23^ and designing a triply resonant cavity is, in principle, achievable. We demonstrated spatial coherence across the array and showed that we can exert full control over the spatial properties and polarization of the output beam through appropriate tailoring of the resonator design and placement. This design can be readily transferred to different wavelengths by changing the quantum well materials (for example III-Nitrides have ISTs at near-infrared frequencies^16^,^26^ while most III–V heterostructures support ISTs in the THz range), and the nanocavity design.

## Methods

### Metamaterial design

The metamaterial was designed using Lumerical FDTD solutions by Lumerical Inc.^21^. The split rings are formed by a Boolean subtraction of two ellipses: the outer ellipse has radii of 480 and 400 nm along the *x*- and *y*-directions; and the inner ellipse has radii of 230 and 300 nm along the *x*- and *y*-directions. The SRR gap width is equal to 125 nm, and the array periodicity is 1.5 μm for both the *x*- and *y*-directions. The SRRs were patterned on the semiconductor heterostructure shown in Supplementary Fig. 2 using standard e-beam lithography followed by deposition of 100 nm Au with a 5 nm Ti adhesion layer and a standard liftoff process.

### Quantum well design and characterization

The quantum well structure was designed using the commercially available Nextnano3 software^22^ implementing the 8-band **k.p** model, which provided the band structure results in Fig. 3a. The fabricated semiconductor heterostructure depicted in Supplementary Fig. 2a was cleaved and polished at a 45° angle. Transmittance in the wedge configuration was measured using a Fourier transform infrared spectrometer (Bruker IFS-6600) as reported in Supplementary Fig. 2b.

### SHG characterization

To measure SH generation, the sample was pumped using a grating tunable, linearly polarized continuous wave CO_2_ laser (for the high-intensity pumping, an optical parametric amplifying system with 12-ns pulses clocked at 1 kHz was used), and the emission at the SH frequency was measured using a calibrated InSb detector. High-numerical aperture aspheric lenses were used to focus (collect) light on (from) the sample. The continuous wave beam was mechanically chopped at 2 kHz and the detector signal was demodulated with a lock-in amplifier. The pump beam diameter was measured to be ∼100 μm in the sample plane. A long pass filter was used before the sample to remove unwanted frequencies from the pump and a MgF_2_ lens was used to refocus the SH signal on the detector and also as a short pass filter to prevent the pump from hitting the detector. As a control experiment, the sample was translated (perpendicular to the beam path to ensure the sample remained in the focal spot of the pump) to a location where no metamaterial was present. At this position no SH signal was detected; this control was repeated for each experiment described.

### Far-field calculations and measurements

To compute the far field of the first case in Fig. 5a we simulate (using an FDTD full-wave simulator) *I*_single_(*θ,ϕ*), the far-field intensity profile of a single resonator; a justification is given in Supplementary Note 5. For the second case we turn to ‘antenna theory'^1^ and multiply the field corresponding to *I*_single_(*θ*,*ϕ*) by the ‘array factor', AF(*θ*,*ϕ*), given by 
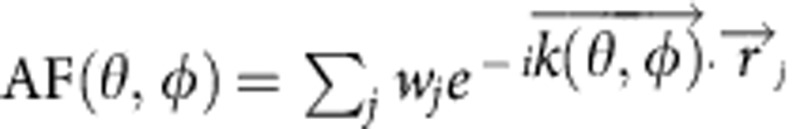
, where *w*_*j*_ and 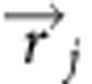 are the weight and position of the *j*th resonator, respectively; 
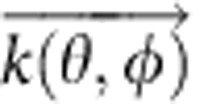
 is the wave vector of the outgoing radiation at the SH frequency and the summation is over all resonators illuminated by the FF pump. The weights in AF are chosen to represent the FF pump by taking the value to have a spatial Gaussian dependence (with a width corresponding to the measured beam waist) on the lateral coordinates of 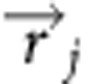. Experimental mapping of the far-field intensity distribution was done by replacing the high-numerical aperture collection lens with a 1′′ diameter, 5 cm focal length ZnSe spherical lens followed by an iris before the 1′′ MgF_2_ lens. We set the iris to ∼2 mm and translate it perpendicular to the beam path while recording the SH signal in several positions. The ZnSe lens maps solid angles to lateral positions so we numerically remap it to solid angles for comparison with the numerical simulation.

### Beam manipulation measurement setup

Experimental mapping of the far-field intensity distribution for large angles required replacing the two-lens imaging system described above with a single MgF_2_ spherical collection lens having a 1′′ diameter and 7.5 cm focal length. This collection lens and the detector were mounted on a rotating arm (movable in the horizontal plane) with the axis positioned beneath the sample. The angular resolution for this configuration is about 10°. For this experiment the incident beam spot size was measured as ∼50 μm due to an addition of a beam-expanding telescope before the high-numerical aperture focusing lens.

## Additional information

**How to cite this article:** Wolf, O. *et al.* Phased-array sources based on nonlinear metamaterial nanocavities. *Nat. Commun.* 6:7667 doi: 10.1038/ncomms8667 (2015).

## Supplementary Material

Supplementary Information Supplementary Figures 1-5, Supplementary Note 1-6 and Supplementary References

## Figures and Tables

**Figure 1:**
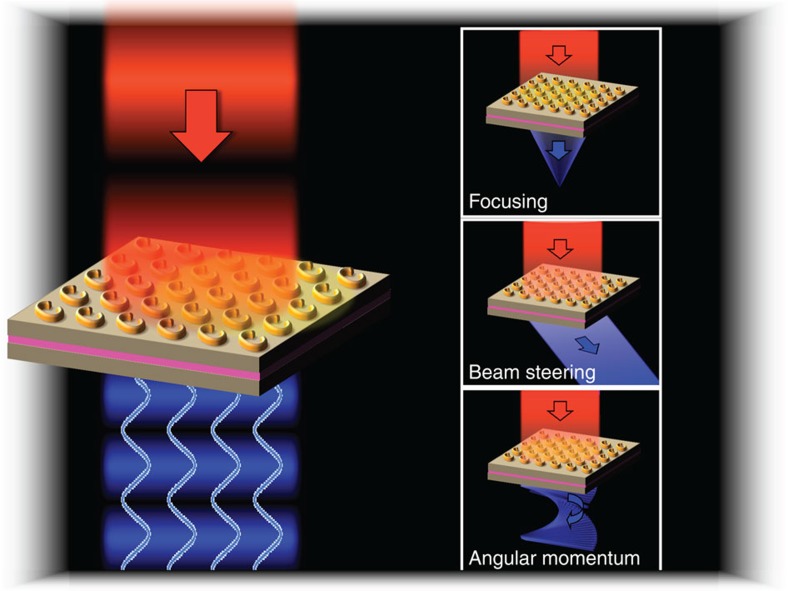
Artist's impression of the ultrathin phased-array device. The figure depicts the SH-generating quantum well layer (pink) and the beam-shaping metamaterial resonators. The phase-coherent emission from the individual resonators opens the door for many beam-shaping applications, some of which are presented in the insets. Each type of device can be achieved through proper design of the individual metamaterial resonators and their arrangement on the surface.
