# Supplementary material for: Phased-array sources based on nonlinear metamaterial nanocavities
Source: Nat Commun. 2015 Jul 1;6:7667. doi: 10.1038/ncomms8667 (PMC4506537; doi:10.1038/ncomms8667)
Supplement: Supplementary Information — Supplementary Figures 1-5, Supplementary Note 1-6 and Supplementary References [file ncomms8667-s1.pdf]

Supplementary Figures

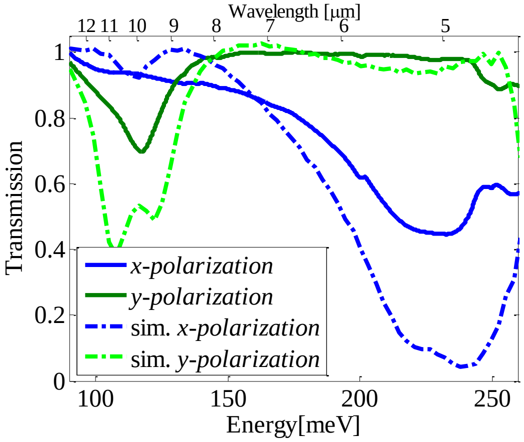

Supplementary Fig. 1 Simulated transmittance (dashed) and normalized FTIR spectra (solid) of the complete sample in both polarizations for normal incidence excitation.

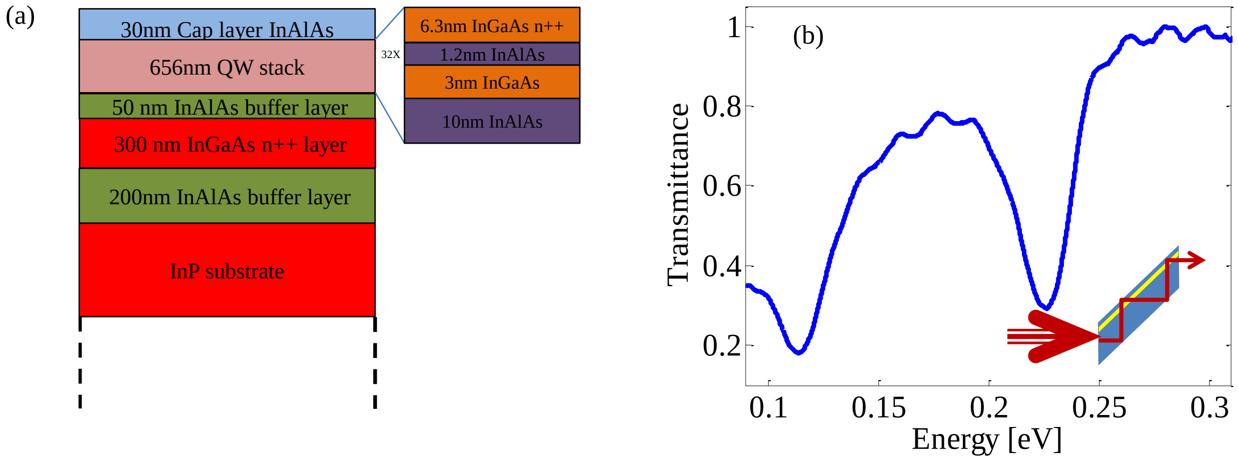

Supplementary Fig. 2. (a) Schematic drawing of the semiconductor heterostructure. The QW layer comprises 32 identical periods shown in the inset. (b) FTIR transmittance spectrum of the grown semiconductor heterostructure measured in a wedge configuration as depicted by the inset. The yellow stripe represents the QW stack. This spectrum was used to design the subwavelength nanoresonators.

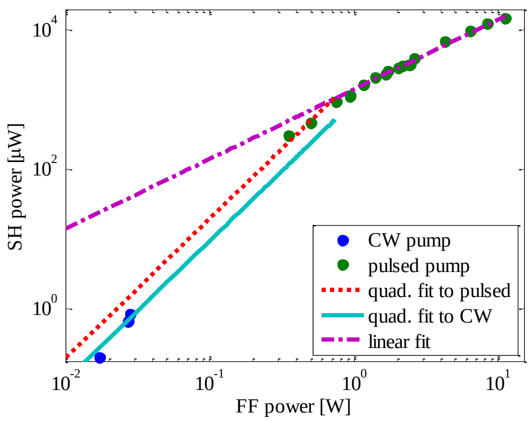

Supplementary Fig. 3. Logarithmic plot of SH signal as a function of pump power. 'Pulsed pump' data points are given for λ=10.1 µm, 'CW pump' data is for λ=10.22 µm for comparison.

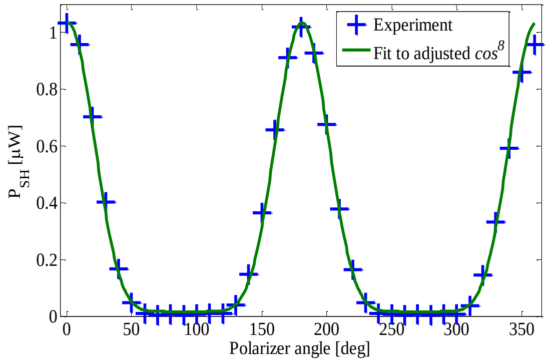

Supplementary Fig. 4. SH signal as a function of pump polarizer angle. 0 degrees corresponds to the pump polarization and the fitting function is adjusted for unintentional sample tilt.

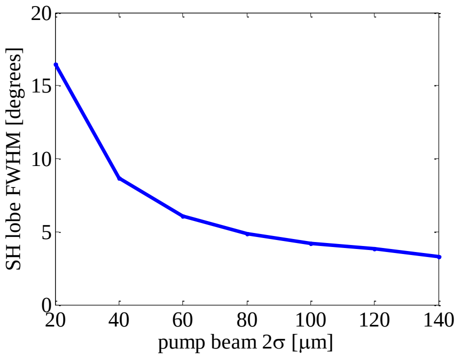

Supplementary Fig. 5. Simulation of expected SH lobe width as a function of pump beam spot size.

Supplementary Notes

Supplementary Note 1: Linear characterization of the complete sample

Supplementary Fig. 1 depicts the normalized FTIR spectra (to the respective maxima) of the complete sample measured at normal incidence for two polarizations of the incoming beam. The figure also reports the full-wave FDTD, simulated transmittance. We observe good qualitative agreement with simulations; the deviations are most likely due to fabrication imperfections. The ISTs in QWs were simulated through an anisotropic permittivity tensor description (see [1] for mathematical expressions) where the in-plane components were the background permittivity of the semiconductor material and the normal component had additional two Lorentzian resonances with center frequency (width) taken from the wedge measurement described below. The values are 27.6(1.8) THz and 54.8(3.3) THz. A comprehensive theoretical analysis of this structure has been presented elsewhere [1].

Supplementary Note 2: Semiconductor heterostructure design and characterization

We design the device for pumping by a CO_2 laser, i.e. at ~10 µm. A suitable material system for this wavelength is the III-As semiconductor system. The semiconductor heterostructure employed in the manuscript is schematically reported in Supplementary Fig. 2(a). It comprises an asymmetric double QW based on the design in [2] supporting three ISTs, two at the FF and one at the SH, resulting in a large second order susceptibility. The QW stack consists of 32 identical periods based on an $In_{0.53}Ga_{0.47}As/Al_{0.48}In_{0.52}As$ QW structure lattice-matched to the InP substrate as shown in the inset of Supplementary Fig. 2(a). The highly doped InGaAs layer below the QW stack does not play any role in the SHG enhancement discussed in this work, because in the frequencies of interest it behaves as a lossy dielectric. We intend to use this layer for electrical measurements in subsequent work. The transmittance spectrum depicted in Supplementary Fig. 2(b), measured in a ‘wedge’ configuration shown in the inset, exhibits two dips that correspond to the FF and SH frequencies, proving the presence of the designed IST absorptions.

Supplementary Note 3: Second harmonic process characterization

The vertical spread of data points appearing in Fig. 4(A) in the manuscript for adjacent frequencies is mainly due to different lasing lines having different spatial mode profiles and therefore a slightly different spot size resulting in a different intensity on the sample. Slight differences in efficiency between the CW and pulsed pumps are expected owing to the very different nature of these sources (i.e., different beam profile, CW versus short pulse), however using this low repetition rate, high peak power source enables us to access excitation powers much higher than those available for our CW setup, this source also spans frequencies not present in a CO_2 laser. In addition to the filters employed in the CW setup, crossed polarizers were used before and after the sample to ensure no signal is leaking from the pump to the detector. In Supplementary Fig. 3 we plot the SH peak power as a function of the FF peak power on a logarithmic scale. The high intensity, pulsed pump data is for the wavelength with the highest efficiency in Fig. 4(A) while the CW data is for a wavelength available in the CO_2 laser and is given for comparison. We note

that in this high power regime we see a cross-over from the quadratic power dependence observed at low powers to a linear behavior. This saturation may arise from the ISTs' absorption saturation, the full resonance case discussed in [3] predicts a saturation of the conversion efficiency (referred to as 'parametric gain' there). Predicting this crossover point can be done using an electrodynamic FDTD simulation that is coupled to spatially-resolved rate-equations for the populations in the QW subbands. In principle, this can provide a good estimation of the crossover point, however it requires knowledge of many parameters relating to the various relaxation mechanisms in the intersubband system. This is out of the scope of this paper. Deviations from a quadratic power dependence due to saturation of IST absorption and harmonic generation were observed before for power densities of 150 kW/cm^2 [4] and our resonators likely concentrate the incident light to these levels.

Supplementary Note 4: Polarization experiments

To verify that the sample responds only to y polarized light we place a polarizer before the sample. Supplementary Fig. 4 shows the result of the input polarization measurement; as can be seen, the data (blue crosses) is well fitted by the expected theoretical curve. As additional confirmation, the sample was rotated 90 degrees, and in this position no SH signal was detected. This serves as confirmation that our sample responds to one polarization only. The fitting function for the above polarization measurement is $A\left(cos(\theta)^2 cos^2(\theta+\varphi)\right)^2 + B$; where, θ is the polarizer angle, φ is a fitting parameter corresponding to an unintentional rotation of the sample, and A and B are also fitting parameters corresponding to the signal amplitude and a non-ideal extinction ratio. Here the values are φ ~ 2 degrees, A ~ 1.02 μW and B ~ 0.02 μW). This functional dependence can be explained as follows. The first cosine term represents the pump power attenuation arising from the polarizer; the second cosine term represents the power impinging on the sample at the correct polarization to excite the resonator. Lastly the additional squaring comes from the quadratic dependence expected for the SH process. The 'analyzer configuration' reported in the main text (Fig. 4(B)) is fitted to $Asin^2(\theta+\varphi) + B$. The parameters here represent the same quantities as in the above case, and for the data presented here the fitting parameters are φ ~ 6.2 degrees, A ~ 0.9 μW and B ~ 0.005 μW.

Supplementary Note 5: Far-field computation

For the first case in Fig. 5(A), since the resonators, which act as sources, are uncorrelated we sum their intensities. Since the spot size of the pump is less than 100 μm and in our experimental setup we are collecting at a distance of ~5 cm, it is reasonable to assume that all cavities are essentially in the same location; this implies the total radiation pattern is equal to the single cavity far-field radiation pattern and that the absolute intensity depends on the number of radiators contributing. For the second case in Fig. 5(B), Supplementary Fig. 5 shows the expected SH beam width as a function of the pump beam spot size assuming the array emits coherently over this size. We notice that in order to get SH beam lobes on the order of a few degrees, the coherence length must be at least comparable to the (~100 μm) spot-size used in the experiment.

From this plot we can also infer the behavior expected for the intermediate case (i.e. the sources are coherent over some length scale that is shorter than the experimentally used beam size). Similar to the first case, the measured intensity for the intermediate case would be an incoherent summation over individual contributions, but each of the individual contributions is now calculated using the second case with a coherence length equal to the beam waist of Supplementary Fig. 5. Such a procedure further confirms that to match the experimentally measured far-field profile, the coherence length must be close to the experimentally used beam size.

Supplementary Note 6: Phase-front control example

To analyze the mirroring effect we consider the resonator as a dipole antenna with an *edge feed*. This is a reasonable approximation because SH radiation is generated mainly in the region of the SRR gap (due to the FF resonance of the SRR) and at that frequency the SRR has a dipolar resonance oriented in the x direction. In this picture it is clear that two, opposite facing, edge fed dipole antennas (being fed in phase) emit radiation with π phase shift. This picture also serves as a guide to designing resonators with arbitrary phases: the 'feed' location must supposedly be moved along the SH dipole's length.

Supplementary References:

1. Campione S, Benz A, Sinclair MB, Capolino F, Brener I. Second harmonic generation from metamaterials strongly coupled to intersubband transitions in quantum wells. *Applied Physics Letters* 2014, **104**(13)**:** 131104.

2. Capasso F, Sirtori C, Cho AY. Coupled quantum well semiconductors with giant electric field tunable nonlinear optical properties in the infrared. *Quantum Electronics, IEEE Journal of* 1994, **30**(5)**:** 1313-1326.

3. DeTemple T, Bahler L, Osmundsen J. <title>Semiclassical theory of resonant three-wave parametric interactions: Second-harmonic generation</title>. *Physical Review A* 1981, **24**(4)**:** 1950-1964.

4. Boucaud P, Rosencher E, Bois P, Julien FH, Yang DD, Lourtioz JM. Saturation of second-harmonic generation in GaAs–AlGaAs asymmetric quantum wells. *Opt Lett* 1991, **16**(4)**:** 199-201.
